# Extreme Heat Exposure in the Construction Industry: A Scoping Review on Risk Factors and Heat-Related Health Consequences

**DOI:** 10.3390/ijerph22111651

**Published:** 2025-10-30

**Authors:** Shaila Nazneen, Sang D. Choi, Gabriel Ibarra-Mejia

**Affiliations:** 1 Environmental Science and Engineering (ESE) Program, The University of Texas at El Paso, El Paso, TX 79968, USA; snazneen@miners.utep.edu; 2 Department of Global and Community Health, College of Public Health, George Mason University, Fairfax, VA 22030, USA; 3 Department of Public Health Science, The University of Texas at El Paso, El Paso, TX 79968, USA; gabmejia@utep.edu

**Keywords:** occupational heat stress, heat-related illness (HRI), construction workers, heat mitigation strategies, workplace safety, rising ambient temperatures

## Abstract

Prolonged exposure to high ambient temperatures, heat stress, and inadequate mitigation measures increases the health and safety risks of construction workers. Following the PRISMA guidelines, our goal was to synthesize recent evidence on the impacts of ambient heat stress on construction workers. A literature review of articles published between 2019 and 2024 was conducted, selecting 42 out of 883 articles that focused on occupational heat stress, extreme ambient temperatures, and their effects on the health, safety, and injury risks of construction workers. The findings highlighted a relationship between occupational heat exposure, increased injury, illness, and mortality among construction workers. Elevated ambient temperatures, during summer and peak work hours, significantly increased the risk of falls, cardiovascular events, and thermal discomfort. Younger, unacclimatized workers in regions with extreme ambient heat and a lack of regulation, observation, and regulation enforcement were at risk. Evidence revealed gaps in worker training, compliance, enforcement, and the integration of individualized monitoring. This review highlights the increasing risks associated with occupational heat exposure in construction workers, driven by rising ambient temperatures. It emphasizes the need for integrated strategies combining personalized wearable technologies, inclusive training, and regulatory reform to improve worker safety and productivity and promote policy development.

## 1. Introduction

Occupational heat stress represents a critical threat to worker safety and productivity across multiple industries, with construction personnel facing disproportionately elevated risks due to the convergence of extreme environmental conditions and physically demanding labor requirements. Heat-related occupational injuries greatly affect the workforce. In 2021 and 2022, there were a total of 5770 reported “Days Away from Work, Job Transfer, or Restriction” (DART) cases due to heat. This averaged 2885 cases per year in the United States alone, with construction accounting for over 35% of these incidents despite representing only 6% of the workforce [1,2,3]. However, the number of heat-related illnesses and injuries among workers may be underestimated. Among outdoor workers, construction personnel represent a particularly vulnerable population due to the combination of uncontrolled thermal environments, sustained physical exertion, and time-sensitive project demands that often preclude adequate heat stress mitigation measures.

The physiological burden imposed by occupational heat exposure operates through well-established thermoregulatory pathways. Singh et al. (2019) demonstrated that combined environmental and metabolic heat stress can overwhelm the body’s cooling mechanisms, leading to core temperature elevation and cardiovascular strain [4]. Games et al. (2020) specifically linked this physiological compromise to increased injury risk through impaired cognitive function, reduced motor coordination, and decreased situational awareness [5]. Construction workers exhibit heightened vulnerability across demographic and experiential spectra, with Calkins et al. (2019) highlighting notably higher injury rates among both younger workers (18–24 years) lacking heat acclimatization and older workers (>54 years) with diminished thermoregulatory capacity [6]. This risk intensifies among workers with limited job experience and those employed by smaller contractors with fewer resources for heat stress prevention programs.

The theoretical framework underlying heat-related injury mechanisms centers on the disruption of thermal homeostasis. When environmental heat gain and metabolic heat production exceed the body’s cooling capacity through evaporation, convection, and radiation, core body temperature rises, triggering a cascade of physiological responses. These responses, while initially protective, can compromise cognitive processing, reaction time, and postural stability factors directly linked to traumatic injury risk, particularly falls from elevation and equipment-related accidents [7,8].

Despite growing recognition of heat stress as a significant occupational hazard, substantial knowledge gaps persist that limit the development of effective, evidence-based prevention strategies. Current research inadequately elucidates the specific physiological and cognitive mechanisms through which thermal stress translates to increased injury risk. Spector et al. (2019) identified this critical gap, noting that while epidemiological evidence demonstrates clear associations between heat exposure and injury rates, the underlying causal pathways remain poorly characterized [9]. This mechanistic uncertainty impedes the development of targeted interventions and limits the precision of risk assessment protocols.

While the Heat-Shield consortium and other research initiatives have proposed various heat stress mitigation strategies, rigorous evaluation of intervention effectiveness remains limited [8]. Systematic assessments of cooling technologies, work–rest scheduling protocols, hydration strategies, and personal protective equipment modifications have been insufficient to establish evidence-based best practices. This gap is particularly pronounced regarding interventions specifically designed for construction work environments, where implementation feasibility and cost-effectiveness considerations are paramount. Current measurement approaches demonstrate a restricted scope, typically capturing personal exposure proxies over extended durations rather than acute exposure events most relevant to injury risk [10]. Moreover, existing studies have inadequately addressed workplace diversity, with limited representation of different construction trades, geographic regions, and demographic subgroups. This representational gap constrains the generalizability of findings and limits the development of inclusive prevention strategies.

The economic and human costs of heat-related occupational injuries demand urgent attention from practitioners, policymakers, and industry stakeholders, as they estimate the economic loss from reduced labor productivity due to heat exposure. The Pennsylvania Compensation Rating Bureau (2024) pointed to nearly USD 968 million in heat-related illness claims between 1999 and 2018. However, we should consider that indirect costs (like productivity losses) could triple the direct cost figure [11]. This is a significant understatement according to the cited source. The primary finding of a study estimated the economic loss from reduced labor productivity due to heat exposure. Their model estimated this indirect cost to be between USD 76 billion and USD 117 billion for the year 2010 [12]. Beyond economic considerations, heat stress contributed to approximately 11 construction worker fatalities per year from 1992 to 2022, representing preventable tragedies that devastate families and communities [13]. Current regulatory frameworks, including OSHA guidelines and state-specific heat illness prevention standards, rely on limited evidence bases that may not reflect the complexity of modern construction environments. An enhanced understanding of exposure–response relationships and intervention effectiveness could inform more precise regulatory standards and industry-specific guidance documents.

This scoping review aims to synthesize the most current evidence (2019–2024) on heat stress, health impacts, and occupational injury risk relationships among construction workers, with particular emphasis on identifying effective prevention and mitigation strategies at the workplace level. Unlike previous reviews that have identified gaps in heat stress assessment, our review uniquely integrates recent findings to (1) inform evidence-based prevention guidelines applicable to current construction practices, (2) provide specific regulatory policy recommendations based on emerging evidence, and (3) establish future research priorities that build upon, rather than simply reiterate previously identified gaps in heat stress assessment approaches.

## 2. Materials and Methods

### 2.1. Exploratory Literature Review

Given that this is a relatively emerging area of study, existing research and publications are scarce in this area. Therefore, the review framework cannot accommodate meta-analysis or systematic reviews, as these methods rely on a large number of publications that consistently follow similar methodologies to establish valid and reliable results. Therefore, we opted to adopt a scoping literature review to emphasize exploring the content of the articles, rather than focusing on statistical measures. This scoping review was registered as an open-ended registration on OSF registries (Registration DOI: https://doi.org/10.17605/OSF.IO/W6VYX). We utilized the PRISMA scoping review standards [14] to ensure the methodological rigor of our study.

### 2.2. Eligibility Criteria

Peer-reviewed articles published in English between 2019 to 2024 were included in this scoping review. Initially, articles and reports on heat stress among construction workers, including clinical studies, clinical trials, clinical trial protocols, controlled clinical trials, randomized controlled trials, comparative studies, observational studies, government publications, guidelines, introductory journal articles, reviews, systematic reviews, meta-analyses, newspaper articles, preprints, technical reports, webcasts, and case reports, that were published from 2019 to 2024, with both abstracts and full texts available for screening, were included in the screening process. Animal trial studies, articles that were published before 2019, and articles published in any language other than English were excluded. First, we kept our eligibility criteria open to explore the extent of the available literature on this topic. After completing the initial search, we updated our eligibility criteria to include only peer-reviewed articles that were published in English. In the second stage of review, i.e., in the screening stage, we only included peer-reviewed articles for further analysis.

### 2.3. Search Strategy

We conducted our search using the following databases: PubMed (National Library of Medicine), Scopus, Web of Science, and ScienceDirect (Elsevier). The initial search included articles published in English between 1 January 2019 and 4 December 2024, with available abstracts and full-text access. The target population was adults aged 19 to 64 years. The article types included in the filter were case reports, clinical studies, clinical trials, clinical trial protocols, comparative studies, controlled clinical trials, English abstracts, government publications, guidelines, introductory journal articles, meta-analyses, newspaper articles, observational studies, preprints, randomized controlled trials, reviews, systematic reviews, technical reports, and webcasts. However, since our primary focus in this review was on recent developments in the field published in peer-reviewed journals, we excluded the gray literature from the final review. Our search term included “construction workers”. Any study that focused on construction workers only or on outdoor workers, along with the other categories, such as agricultural and mining sector construction workers, was also included in the original research. We included those studies in our final analysis, extracting the information only for the construction workers as much as possible. A total of 883 articles were identified through the search conducted on 10 December 2024, in all four databases. The final search terms for each database included heat stress, heat exposure, occupational heat stress, thermal stress, heat-related illness, heat strain, construction, construction workers, postural balance, fall risk, occupational injuries, workplace safety, temperature, heat, and extreme heat. The keywords, Boolean operators, and combinations used across four databases are provided below.

PubMed (755 articles)

(heat stress*) OR (heat exposure*)) OR (occupational heat stress*)) OR (thermal stress*)) OR (heat-related illness*)) OR (heat strain)) AND (construction)) OR (construction workers*)) AND (postural balance)) OR (fall risk*)) OR (occupational injuries*)) OR (workplace safety*)) OR (workplace safety *)) AND (temperature)) OR (heat)) OR (extreme heat*).

Scopus (75 articles)

((“heat stress” OR “heat exposure” OR “occupational heat stress” OR “thermal stress” OR “heat-related illness” OR “heat strain”) AND (“construction” OR “construction workers” OR “roofers” OR “manual workers” OR “outdoor workers”) AND (“occupational injuries” OR “workplace safety” OR “workplace risk factors”) AND (“temperature” OR “heat” OR “extreme heat” OR “high temperature”)).

Web of Science (40 articles)

TS = (heat stress* OR heat exposure* OR occupational heat stress* OR thermal stress* OR heat-related illness* OR heat strain) AND TS = (construction OR construction workers*) AND TS = (postural balance OR fall risk* OR occupational injuries* OR workplace safety* OR workplace risk factors*) AND TS = (temperature OR heat OR extreme heat*).

Science Direct (13 articles)

Heat stress/exposure/strain/thermal stress/occupational heat stress and fall risk/postural balance/occupational injuries and workplace safety/risk factors among construction workers in extreme heat/heat/high temperature.

### 2.4. Review Strategy

**Figure 1:**
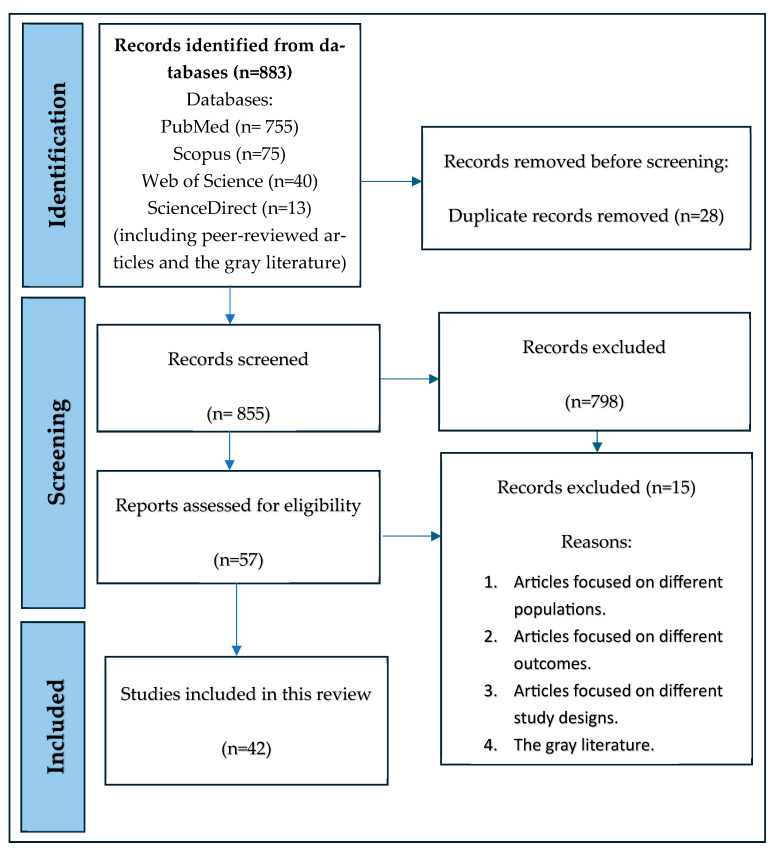
Review process flowchart illustrating the study selection results, following the PRISMA (Preferred Reporting Items for Systematic Reviews and Meta-Analyses) guidelines.

The screening procedure was conducted using a two-phase decision-making approach. In the first phase, we utilized Rayyan [15], a web-based tool designed to facilitate the organization and execution of literature reviews. The search results from all databases, consisting of titles, abstracts, and full texts, were extracted and then imported into Rayyan for screening. Next, all the authors evaluated each article/report based on the inclusion criteria. After reviewing all the abstracts and systematically resolving the differences of opinion, a total of 57 articles were finally selected for a full-text review. Finally, 42 full papers were considered for the final analysis. Figure 1 portrays a detailed overview of the records collected from individual databases. 

A data matrix was developed for systematically extracting relevant data from the included studies. Independent data extraction was conducted by all the authors, and a weekly team meeting was organized to discuss and compare the data matrix among all authors. Due to inconsistencies in the methodological approaches followed by the included studies and their reporting of findings, conducting any statistical analysis was not feasible, although we reported the frequencies of different subclasses.

## 3. Results

### 3.1. Selection of Studies

Among the 883 articles, Rayyan identified 66 duplicates. Rayyan deleted 28 duplicated articles, leaving 855 articles to be screened. After a thorough review of the titles and abstracts, 798 articles were excluded, and a total of 57 articles were selected for full-text screening. After a full-text review of all 57 articles, a total of 42 peer-reviewed articles were included in the final analysis.

### 3.2. Year of Publication and Country of Origin

Table 1 shows the absolute and relative frequencies of the finally included article characteristics in our scoping review. As shown in Table 1, in 2019, a total of 11 articles (26.2%) were published on this topic, demonstrating substantial scholarly attention. The publication frequency then decreased in subsequent years until 2024, when there was a notable increase, with nine (21.43%) publications on this subject. The majority of the studies were conducted in the United States (10 studies).

From Table 1, we can identify that the most common research methods included 21 observational studies, e.g., [16,17,18,19], and 8 systematic reviews and meta-analyses, e.g., [9,20,21,22]. The cross-sectional studies often employed a logistic regression model to analyze the data. Several other articles employed a combined technique of systematic review and meta-analysis and conducted an observational study, which involved collecting primary data at a single point in time from diverse construction worker groups and analyzing combined results statistically to generalize the study findings. Many studies utilized meta-analysis methods in combination with systematic reviews, indicating a common approach to synthesizing findings from multiple observational studies.

Table 2 summarizes the characteristics of studies included in this review. Among the analyzed papers, several key insights and patterns emerged evidently. The topic most extensively studied was temperature/extreme heat (addressed in 36 articles, e.g., [16,18,20,23]), heat stress/heat strain/thermal stress (addressed in 30 articles, e.g., [23,24,25,26]), wet-bulb globe temperature (WBGT) (addressed in 10 articles, e.g., [27,28,29,30]), humidity/relative humidity (addressed in 10 articles, e.g., [27,28,31,32,33]), occupational heat stress/illness/injuries/accidents (addressed in 28 articles, e.g., [9,16,20,34,35]), heat-related illness (addressed in 33 articles, e.g., [16,17,18,23,36,37]), and construction workers and workplace safety (addressed in 40 articles, e.g., [1,38,39,40]). This highlights a broad academic acknowledgment of extreme temperatures as a critical factor influencing occupational safety and the health of the workers in the construction industry. As mentioned above, several studies reported productivity losses among construction workers when WBGT exceeded 28 °C and above.

As shown in Table 2, along with weather determinants, individual and physiological factors, such as age (addressed in 10 articles, e.g., [1,17,28,29]), heart rate (addressed in 10 articles, e.g., [24,25,26]), and skin/body/oral/auditory canal temperature (addressed in 9 articles, e.g., [28,41,42,43]), were also considered, especially by the observational studies conducted. Conversely, gender (explored in four articles, e.g., [9,20,44]), blood pressure (explored in three articles [29,32,45]), and urinary albumin–creatinine ratio (ACR) (studied in just one paper [37]) were the least explored topics, indicating potential research gaps that need further investigation.

Overall, the research designs of the 42 reviewed studies varied widely based on study objectives and data availability, ranging from simple observational analyses to complex spatial–temporal models. The methods varied significantly, including case–crossover designs with logistic regression, time-series regression models (e.g., Poisson and quasi-Poisson models), field-based observational studies employing environmental and physiological monitoring, GIS-based spatial analysis, the utilization of big data analytics, and experimental and quasi-experimental studies with controlled settings.

**Table 2:** Characteristics of the studies included in this review, including short summaries.

Title	First Author/Year of Publication	Target Group	Heat-Related Risk Factors	Findings	Recommendations/Interventions
Heat exposure and productivity loss among construction workers: a meta-analysis	[46]	A total of 2387 construction workers across 14 cross-sectional studies in India, China, Iran, Thailand, Australia, Italy, Saudi Arabia, and Sweden	Heat exposure. Wet-bulb globe temperature (WBGT). Construction/construction workers. Worker age and gender. Economic context. Productibility loss.	Overall, 60% of construction workers faced heat-related productivity loss, especially above 28 °C WBGT or 35 °C temperature, with older workers (≥38 years), female/mixed-gender teams. High-income countries showed slightly higher loss rates.	Heat adaptation strategies in the construction sector.
Effects of Heat Stress on Workers’ Physical Fatigue and Attentiveness: Multimodal Roofing XR Simulation	[24]	A total of 30 participants (22 males and 8 females) in Indiana, USA	Heat exposure. Heat stress/heat strain. Construction/construction workers. Heart rate. Perceived exertion.	Elevated heat levels significantly increased physical fatigue and reduced workers’ hazard detection ability, raising the risk of accidents under heat stress.	Practical interventions to reduce heat-related risks and improve safety in construction settings.
Cooler break areas: Reducing heat stress among construction workers in Japan	[45]	A total of 26 male rebar workers; 196 observations collected, 152 used after data exclusion from five construction sites located in urban areas of Japan	Ambient temperature. Humidity. Pulse rate. Blood pressure. Forehead skin temperature. Step counts. Self-reported fatigue.	Cooler break environments significantly reduced physiological strain, showing lower pulse rates, decreased forehead temperatures, and stabilized blood pressure after breaks. Workers felt more subjectively tired afterward, indicating a possible psychological trade-off.	Management of temperature differences is essential to ensure both physiological benefits and psychological comfort.
Association between temperature and occupational injuries in Spain: The role of contextual factors in workers’ adaptation	[44]	Over 22.3 million occupational injuries in 48 provinces in mainland Spain and the Balearic Islands (excluding the Canary Islands, Ceuta, and Melilla)	Temperature/extreme heat. Occupational injuries. Age. Sex. Economic sector. Educational attainment. Unemployment rates. GDP. Employment conditions.	U-shaped link between temperature and occupational injuries, with heat posing greater injury risks. Young men, workers in agriculture, construction, and hospitality were vulnerable to heat. Cold affected women, older workers, and service workers more.	Particularly for vulnerable sectors, younger and less educated workers, and regions with extreme temperatures.
Extreme Heat and Occupational Health Risks	[21]	Multiple studies	Heat-related illness. Workplace Safety. Ambient temperatures. Physical exertion. Use of personal protective equipment. Socioeconomic vulnerabilities.	Agriculture, construction, military, firefighting, mining, and manufacturing were identified as high-risk industries for HRI, with workers in LMICs disproportionately affected due to limited cooling and protections.	Need for robust heat stress prevention programs, regulatory policies, workplace measures, and continued research to tackle the rising impact of occupational HRI intensified by climate change.
Exploring the Influence of Extreme Weather on Construction Worker Safety	[47]	A total of 12,624 construction accidents	Temperature/extreme heat. Extreme weather event (e.g., heat, flood, hail, wildfire). Occupational injuries (e.g., falls, heat-related illnesses). Temporal variations across years and months. Construction workers.	Extreme heat, floods, hail, and wildfires—especially wildfires—increased construction accident risk, while cold storms, strong winds, and snow reduced it. Falls and heat-related illnesses were more common during extreme weather events.	Tailored safety measures and proactive planning are essential.
Real-time risk assessment of multi-parameter induced fall accidents at construction sites	[48]	A total of 1718 causalities in Banqiao District, New Taipei City, Taiwan	Heat stress/heat strain. Construction workers. Hazardous work locations. Environmental heat stress (WBGT). Metabolic heat load (HR sensors). Worker acclimatization status.	High temperature and heat strain increase fall risk, especially in summer and high-risk areas. A new risk assessment model successfully identified vulnerable workers and recommended safety measures.	Continuous monitoring of both physiological strain and environmental factors would help prevent workplace falls and injuries.
Mandated Rest Breaks and Occupational Injuries and Illnesses in Dallas County, Texas Construction Workers	[49]	Compensation claims data from 2013 to 2018 in Dallas, Texas	Temperature trends Pre- and post-ordinance occupational illness and injury rates. Construction workers. Construction employment levels	Dallas’s rest break ordinance was linked to slightly lower injury and illness rates compared to Tarrant County, though not statistically significant, possibly due to pre-existing downward trends.	Although the rest break policy may have helped enhance worker safety, more robust and comprehensive regulations are warranted.
Automation in Construction: Spatio-temporal heat risk analysis in construction: Digital twin-enabled monitoring	[31]	For geometric data, 300 UAV images were collected; additionally, weather data were recorded hourly from two weather stations in Stephenville, Texas	Temperature/extreme heat. Heat stress/heat strain. Spatial variations. Relative humidity. Solar irradiance. Wind speed. Surface material properties. Construction workers.	The proposed framework delivered more accurate and timely heat risk assessments than traditional tools like the black-globe thermometer.	Practical guidance for creating effective heat mitigation measures to improve safety in the construction industry was provided.
A case report of near-missed heat stroke	[50]	A case report of a 37-year-old male construction worker in Sarawak, Malaysia, with a history of near-missed diagnosis of exertional heat stroke	Heat exposure.	A patient misdiagnosed with neurological conditions was later found to have heat stroke with rhabdomyolysis. Timely supportive care led to full recovery.	Serum creatine kinase can be a valuable initial screening test in comatose patients.
Development of a Prototype Observatory of Heat-Related Occupational Illnesses and Injuries through the Collection of Information from the Italian Press, as Part of the WORKLIMATE Project	[16]	A total of 35 documented cases of heat-related occupational illnesses and injuries utilizing a media-based surveillance approach	Temperature/extreme heat. Heat-related illness. Occupational injuries. Workplace safety.	In 2022, 57.1% of heat-related cases occurred, with 31.4% in July, mainly among outdoor construction workers during moderate to strong heat stress, according to the Universal Thermal Climate Index (UTCI). Media reports proved useful for occupational health surveillance in increasing heatwaves.	enhance awareness and implement effective prevention strategies to safeguard workers from heat-related hazards
The burden of occupational injury attributable to high temperatures in Australia, 2014–19: a retrospective observational study	[51]	Included data from the Australian workforce (10,669,078 employed workers as per 2016 census data) focusing on disability-adjusted life years (DALYs) lost due to occupational injuries	Temperature/extreme heat. Occupational injuries. Climate zones. Temperature variations. State and territory locations. Demographic and industry-related factors.	Around 2.3% of occupational injuries, totaling 967 DALYs, were associated with high temperatures, with the greatest impact seen in tropical regions and states, such as New South Wales and Queensland. A growing risk of work-related injuries due to rising temperatures.	Adaptive strategies and safety policies customized to local climate conditions and high-risk sectors like agriculture, transportation, and construction.
Proximity Activity Intensity Identification System in Hot and Humid Weather Conditions: Development and Implementation	[27]	Not explicitly stated; data comprised 94,808 frames of video footage with approximately 95,303 worker detection records	Heat stress/heat strain. Temperature. Relative humidity. Wet-bulb globe temperature (WBGT). Construction/construction workers. Worker postures. Workplace safety. Activity intensity (AI). Safety status. Cumulative fatigue of construction crews.	Most construction tasks were moderately intense, heightening heat stress and fatigue. Strong relationship to temperature, sunlight exposure, and elevated AI levels.	Introduction of an innovative automated method for evaluating AI levels. Real-time monitoring will enhance worker safety and productivity in extreme weather conditions.
Solar installation occupational risks: A systematic review	[22]	A total of 31 articles	Temperature/extreme heat. Heat stress/heat strain. Heat-related illness. Four main categories of occupational risks: electrical and fire hazards, heat stress, manual handling, and fall risk. Construction/construction workers. Workplace safety.	Electrical and fire risks were widely studied. Heat stress, falls, and musculoskeletal injuries were under-researched. Gaps in ergonomics, fall protection, and heat stress management for photovoltaic (PV) solar system installers.	Future research can focus on ergonomic evaluations, better safety device design, and enhanced worker training.
Association between extreme temperature exposure and occupational injuries among construction workers in Italy: An analysis of risk factors	[52]	A total of 184,936 construction occupational injuries among construction workers in Italy from 2014 to 2019	Temperature. Occupational injuries. Construction workers. Age. Profession. Working environment. Physical activity. Contact mode.	Elevated temperatures raised injury risk (RR: 1.216), especially for unskilled workers, masons, and those using hand tools or machinery. Colder temperatures offered some protection (RR: 0.901). Heat waves increased risk, with stronger effects at higher temperature thresholds.	Preventive policies to reduce heat exposure risks are essential.
Towards real-time thermal stress prediction systems for workers	[41]	Varied across reviewed studies	Thermal stress. Heart rate. Skin temperature. Core body temperature.	Traditional heat stress assessment methods like thermal indices (WBGT and PSI) had limitations. ECG-based wearable technologies, such as smart garments, chest straps, and patches. offered more accurate real time monitoring of physiological responses. Wearable technologies faced challenges, such as sensor integration, motion-related signal instability, and difficulty in accurately measuring core body temperature.	Further technological advancements to enhance heat stress prevention in the workplace.
Impacts of hot climatic conditions on work, health, and safety in Australia: A case study of policies in practice in the construction industry	[38]	A large construction company and five of its subcontractors in Australia	Construction workers. Workplace safety.	Major policy gaps, including poor worker consultation, weak risk assessments, limited training, and overreliance on administrative controls and PPE. Key areas like heat acclimatization, detailed assessments, and regular policy evaluations were largely missing.	Enhancing workplace heat policies by improving readiness for hot weather, involving workers in decision-making, and integrating thorough risk assessments, training programs, and regular policy evaluations.
Heart rate increase from rest as an early sign of heat-related illness risk in construction workers	[17]	A total of 79 male construction workers from two construction sites in Japan	Temperature/extreme heat. Heat-related illness. Construction/construction workers. Heart rate. Skin temperature. Energy expenditure. Work shifts using physiological sensors. WBGT. Ambient temperature. Humidity. Age. Work experience. Daily physical condition.	A total of 3163 person-time observations were analyzed. Rising heart rate from resting levels indicated early heat-related illness (HRI) risk, with higher WBGT linked to increased HR.	Wearable sensors are effective for monitoring heat stress and enabling timely interventions to protect construction workers.
Best practices used by contractors to reduce heat-related injuries on construction sites	[36]	A total of 46 respondents out of 120 contractors surveyed in Nevada, USA	Heat-related illness. Construction/construction workers. Workplace safety.	Over half (55.56%) of the respondents reported no heat-related illnesses at their sites in the past five years. Overall, 24.44% noted cases, mainly heat exhaustion. Common prevention strategies included the following: Regular hydration (30%); Shaded rest areas (21%). Scheduling work during cooler times of the day (19%).	Comprehensive safety plans, stronger workplace safety laws, and public awareness campaigns. Effective HRI strategies and wider implementation and awareness to improve safety in high temperature environments.
Developing a Geospatial Framework for Severe Occupational Injuries Using Moran’s I and Getis-Ord Gi Statistics for Southeastern United States	[34]	Nearly 50,000 incident records in Southeastern United States	Occupational injuries. Construction/construction workers. Workplace safety.	Southern and southeastern Region 4, especially Alabama counties like Lee, Montgomery, Mobile, and Madison, had the most heat days and highest heat-related injury rates. Nationally, the incidence rate rose sharply from 0.03 per 10,000 employees in 2017 to 0.28 in 2018.	Heat Stress Index (HSI) to routinely assess jobsite heat stress is recommended. Understanding of regional heat-related risks is vital for implementing effective workplace interventions.
Potential Impacts of Different Occupational Outdoor Heat Exposure Thresholds among Washington State Crop and Construction Workers and Implications for Other Jurisdictions	[53]	Entire state population from 39 counties, Washington state, USA	Temperature/heat exposure/daily maximum temperatures. Construction workers. Monthly estimates from the Bureau of Labor Statistics. Employment days at risk of excessive heat exposure.	Differences in temperature exceedances across counties, with Central Washington, especially Yakima County, bearing the highest heat burdens in July and August. Significantly affected crop workers’ exposure above heat thresholds.	Region-specific policies to effectively protect outdoor workers.
Extreme heat and occupational injuries in different climate zones: A systematic review and meta-analysis of epidemiological evidence	[20]	A total of 24 epidemiological studies representing nearly 22 million occupational injury cases in six countries: Australia, Canada, China, Italy, Spain, and the USA	Temperature/extreme heat and occupational injuries (OIs). Industry type (e.g., agriculture, construction, manufacturing). Work environment (indoor/outdoor). Regional climate classifications. Age. Gender.	A 1 °C rise in temperature was associated with a 1% increase in OI risk. Heatwave periods elevated OI risk by 17.4%. Younger male workers and those in physically demanding, outdoor industries were most vulnerable.	Climate and work-specific interventions, particularly in humid subtropical and high-risk oceanic climate zones.
Perceptions of heat-health impacts and the effects of knowledge and preventive actions by outdoor workers in Hanoi, Vietnam	[18]	Cross-sectional Knowledge, Attitudes, and Practices (KAP) survey of 1027 outdoor workers in Hanoi, Vietnam	Temperature/extreme heat. Heat-related illness. Workplace safety. Occupational roles. Awareness of heat symptoms. Access to cooling resources. Hydration practices. Use of weather forecasts.	Many workers reported symptoms like fatigue, headaches, and dizziness but lacked awareness of heat-related illnesses. Construction workers had lower knowledge yet were more likely to seek medical care than street vendors.	Improving awareness of heat-related risks and encouraging preventive measures are essential to reduce heat-related health problems among vulnerable outdoor workers.
Heat-related illness risk and associated personal and environmental factors of construction workers during work in summer	[28]	A total of 61 construction workers (35 at Site 1 and 26 at Site 2) wearing sensors that continuously recording vital signs	Temperature/extreme heat. Heat stress/heat strain. Heat-related illness. Wet-bulb globe temperature (WBGT). Dry bulb temperature Humidity. Construction workers. Heart rate. Skin temperature. Energy consumption. Age. Medical history. Career duration and daily working conditions.	Older age, high skin temperature, elevated post-warm-up heart rate, work location, and limited experience increased HRI risk. Post-warm-up heart rate may indicate morning fatigue, suggesting monitoring could aid prevention.	Continuous monitoring of environmental and personal conditions to effectively manage and mitigate heat-related illness risks in construction settings.
Understanding occupational heat exposure in the United States and proposing a quantifying stress index	[39]	Included all reported heat-related injury and illness incidents from 39 states, with specific county-level analysis within Alabama	Temperature/extreme heat. Heat-related illness. Construction workers and workplace safety. Heat index thresholds. Geographical distribution. Industry type. Temporal trends.	Highest rates of heat-related injuries and illnesses in southern states like Alabama and Florida, with construction workers most at risk. Rising heat-related fatalities indicate existing safety measures are insufficient.	New comprehensive Heat Stress Index (HSI) that integrates environmental and physiological indicators, enabling employers to more effectively manage workplace heat-related risks was recommended.
Climate Warming and Occupational Heat and Hot Environment Standards in Thailand	[42]	A total of 168; 90 construction workers (working outdoors) and 78 foundry workers (working indoors) in 18 construction sites in northeastern Thailand	Occupational heat stress. Workplace wet-bulb globe temperature (WBGT). Relative humidity. Wind velocity. Heat-related symptoms. Construction workers. Heart rate (HR). Auditory canal temperature (Tac).	Only 55% of workers were in heat-compliant workplaces, with noncompliance doubling the risk of unsafe body temperatures and heat symptoms. Gaps in Thai heat exposure laws, including unregulated work–rest cycles and lack of recognition for outdoor heat risks.	Thailand’s heat exposure standards require updates, especially regarding the following: Enforcement; Structured work/rest schedules. Acknowledging heat risks in outdoor jobs, such as construction.
Detailed thermal indicators analysis based on outdoor thermal comfort indices in construction sites in South China	[32]	A total of 1063 male workers in Guangzhou, China	Globe temperature. Mean radiant temperature. Extreme heat. Humidity. Wind speed. Occupational heat stress/heat strain. Heat-related illness. Construction workers. Heart rate. Blood pressure and auditory canal temperature.	Over 95% of workers experienced significant heat stress, feeling “hot” or “very hot.” Wind speeds above 1.3 m/s improved comfort, while temperatures over 34 °C worsened it.	Revised classification of thermal indices is proposed to better evaluate outdoor work conditions.
Heat-health vulnerability in temperate climates: lessons and response options from Ireland	[23]	Included 15 papers	Temperature/extreme heat. Heat stress/heat strain. Heat-related illness.	The key vulnerable groups identified included the following: Older adults; Infants; Pregnant women; Outdoor workers; People with chronic illnesses; Low-income urban dwellers; Those with mental health conditions. Multi-sectoral risks of rising temperatures including food systems and the healthcare sector. Scarce national research and policy on heat-health in Ireland.	Adopting a “health and climate change in all policies” approach to protect vulnerable populations.
A Field Evaluation of Construction Workers’ Activity, Hydration Status, and Heat Strain in the Extreme Summer Heat of Saudi Arabia	[25]	A total of 23 male Indian construction workers (plasterers, tilers, laborers) were monitored and surveys for 260 person-days	Heat exposure. Heat stress/heat strain. Construction/construction workers. Heart rate responses. Hydration status. Physical workload in both indoor and outdoor settings.	Wet-bulb globe temperature-based occupational exposure limits (WBGTOELs) were exceeded on 44% of indoor and 78% of outdoor person-days, indicating substantial heat stress. High heart rate reserve (HRR ≥30%) and dehydration were common. Workers began and ended shifts dehydrated.	Self-pacing work can reduce cardiovascular strain, as it may help mitigate heat-related risks in occupational settings.
Risk of Kidney Injury among Construction Workers Exposed to Heat Stress: A Longitudinal Study from Saudi Arabia	[37]	Four construction sites and 65 construction workers in Al-Ahsa Province, Southeastern Saudi Arabia	Heat-related illness. Occupational heat stress. Construction workers. Measured kidney injury using the urinary albumin–creatinine ratio (ACR) from urine samples, hydration status, sleep duration, body mass index (BMI), obesity, diabetes, hypertension, and shift length.	By the end of summer, 18% of workers showed elevated ACR levels, indicating potential kidney injury. Dehydration, insufficient sleep (less than 8 h per day), and obesity were significantly associated with increased kidney injury.	Ensuring proper hydration, limiting work hours, and encouraging healthy sleep practices to reduce the risk of kidney injury among workers exposed to occupational heat stress.
Air temperatures and occupational injuries in the construction industries: a report from Northern Italy (2000–2013)	[33]	A total of 14,072 injury cases were included in Trento, Italy	Temperature/extreme heat. Occupational injuries. Construction workers. Humidity. Wind speed. Solar irradiation. Injury-related factors. Age. Sex. Injury type Prognosis. Mechanism.	Temperatures ≥35 °C significantly raised injury risk, especially during the first two days of heatwaves and among workers under 40. Cold days showed reduced injury risk, likely due to adaptive behaviors.	Implementation of policies to reduce heat-related risks, particularly for construction workers. Improved training, stronger safety protocols, and adjusted work schedules to prevent temperature-related injuries.
Heat exposure and occupational injuries: Review of the literature and implications	[9]	Varied	Heat exposure. Occupational injuries. Workplace safety. Humidity. Industry type (e.g., construction, agriculture, and smelting). Age. Gender. Geographic contexts primarily in industrialized countries.	Increased heat exposure significantly raised occupational injury risks, especially for young males in agriculture and construction industry. It also stated that injury patterns varied by industry, where heat-related cognitive and physical impairments were the key mechanisms.	Transdisciplinary collaboration and inclusive stakeholder engagement amid growing climate change challenges.
Evaluation of the impact of heat stress on the occurrence of occupational injuries: Meta-analysis of observational studies	[35]	Meta-analysis included eight studies (five time-series and three case-crossover designs)	Temperature/extreme heat. Occupational injuries. Construction/construction workers.	A significant increase in injury risk was associated with higher temperatures, with a combined risk estimate of 1.005 (95% CI: 1.001–1.009). Subgroup analyses suggested younger workers (under 25 years), males, and those in agriculture, might be more susceptible to heat-related injuries, which was statistically non-significant.	Targeted occupational health policies, and preventive measures. Research to understand the mechanisms linking heat exposure to occupational injuries.
Assessment of Thermal Exposure Level among Construction Workers in UAE using WBGT, HSI and TW L Indices	[54]	A total of 200 construction workers across three site locations in the United Arab Emirates	Air temperature. Humidity radiant heat. Airspeed. Workload intensity. Clothing. Wet-bulb globe temperature (WBGT) index. Heat Stress Index (HSI). Thermal Work Limit (TWL) Measurements were conducted during peak heat hours (12 PM to 3 PM).	WBGT exceeded recommended Threshold Limit Values (TLVs), putting workers at heat stress risk. Only fit, acclimatized individuals could safely work. TWL advised against unacclimatized or solo work. Radiant heat was the main contributor to thermal stress, surpassing metabolic and convective loads.	Shifting work to cooler times (early morning or after sunset). Offering shaded rest areas. Maintaining proper hydration for workers.
HeatWaves Occurrence and Outdoor Workers’ Self-assessment of Heat Stress in Slovenia and Greece	[19]	A climatological analysis of heat wave trends from 1981 to 2017 combined with a cross-sectional study among 286 outdoor workers (216 from Slovenia, 70 from Greece)	Temperature/extreme heat. Heat stress/heat strain. Productivity loss.	Most workers in Slovenia (71–74%) and Greece (60–69%) reported heat stress impacting productivity (71% and 69%) and well-being (74% and 60%), respectively. Common symptoms were thirst, excessive sweating, exhaustion, and headaches. Coping strategies included increased hydration, schedule adjustments, and frequent breaks.	Educational campaigns and effective mitigation strategies to safeguard outdoor workers.
Workplace Heat Exposure Management in Indian Construction Workers Using Cooling Garment	[26]	A total of 29 male construction workers Construction worksite in Ahmedabad city, Gujarat, India	Temperature/extreme heat. Heat stress/heat strain. Construction workers. Heart rate. Oral temperature. Weighted skin temperature. Torso skin temperature. Sweat loss. Subjective responses related to comfort, thermal sensation, and sweating.	Wearing personal cooling garments (PCGs) significantly lowered torso skin temperature, sweat loss, and heart rate. It also improved comfort and reduced thermal strain compared to regular clothing.	Intervention conclusion: PCG offers a cost-effective, efficient, and practical approach for managing heat exposure in outdoor workplaces, particularly in high-temperature environments like construction sites.
Assessment of Heat Stress Exposure among Construction Workers in the Hot Desert Climate of Saudi Arabia	[30]	Ten residential construction sites	Temperature/extreme heat Heat stress/heat strain. Construction workers, workload intensity, and workplace safety. Humidity, wind speed, solar radiation). Compliance with Saudi Arabia’s midday outdoor work ban. Wet-bulb globe temperature (WBGT), Heat Index (HI) and Humidex (HD).	Workers faced frequent heat stress beyond safety limits. Midday bans were insufficient. HI proved a reliable indicator of WBGT-related risk.	Urgent necessity for enhanced heat-protection strategies and guidelines.
Investigation on heat stress of construction workers in summer in Chongqing, China	[29]	A total of 60 construction workers	Temperature/extreme heat. Solar exposure. Heat stress/heat strain. Construction workers. Wet-bulb globe temperature (WBGT). Age. Type of work. Heart rate. Blood pressure. Body temperature. Hydration. Resting habits.	Average effective WBGT (35.8 °C) exceeded recommended limits, indicating severe heat stress. Outdoor workers, such as piling rig operators and scaffolders, experienced elevated metabolic and heart rates.	Implementation of customized protective strategies and monitoring for workers at greater risk of heat stress.
Heat-related deaths among construction workers in the United States	[1]	A total of 285 heat-related deaths among construction workers in Northeast, Midwest, West, and South regions in USA	Temperature/extreme heat. Heat stress/heat strain. Heat-related illness. Occupational injuries. Construction workers. Age. Race/ethnicity. Birthplace. Employment status. Occupation. Geographic region.	Construction workers, though only 6% of the workforce, made up 36% of heat-related occupational deaths, with fatalities rising alongside summer temperatures. Hispanic workers, particularly Mexican-born, and trades like cement masons, roofers, helpers, and brick masons faced significantly higher death risks.	Targeted workplace interventions, stronger regulations, and comprehensive climate change mitigation strategies.
A case-crossover study of heat exposure and injury risk among outdoor construction workers in Washington State	[6]	A total of 63,720 occupational traumatic injury at outdoor construction worksites throughout Washington State	Heat exposure. Occupational injuries. Construction workers. Age. Job experience. Employer size. Time of injury. Geographic region. Injury type.	Injury risk increased with rising heat exposure (humidex), particularly among the following: Younger and older workers; Less experienced; Small employers; Early-day injuries; Lower extremity injuries; Injuries that took place in Western Washington.	Targeted injury prevention strategies and increased heat awareness within the construction industry.
Nationwide epidemiological study for estimating the effect of extreme outdoor temperature on occupational injuries in Italy	[43]	A total of 2,277,432 occupational injuries across 8090 municipalities in Italy	Occupational injuries. Construction workers. Heart rate. Skin temperature. Core body temperature.	Traditional heat stress measures like WBGT and PSI have limitations. Wearable technologies such as ECG-based devices offer accurate and real-time monitoring. For tracking physiological responses, ECG sensors were most reliable.	Improved technology for effective heat stress prevention in workplaces.
Heat Stress Impacts on Cardiac Mortality in Nepali Migrant Workers in Qatar	[40]	Mortality data of approximately 120,000+ Nepali Migrant Workers in Qatar	Temperature/extreme heat. Wet-bulb globe temperature (WBGT). Heat stress/heat strain. Construction workers. Workplace safety. Working conditions. Causes of worker mortality.	Significant correlation between higher WBGT heat exposure and increased cardiovascular mortality. Estimated that around 200 of 571 deaths could have been prevented with proper heat protection.	Stronger occupational health policies and targeted interventions to reduce heat stress risks among migrant workers.

Table 3 displays the key risk factors discussed in the included studies. The factors were grouped into four key categories, including weather factors, physiological factors, individual factors, and workplace-related factors. The numbers indicate the number of studies that assessed each of the abovementioned factors.

#### 3.2.1. Temperature/Extreme Heat

Numerous studies (36 articles, e.g., [16,18,20,23]) highlighted a strong correlation between high temperatures and occupational injuries, particularly in sectors such as agriculture, construction, and manufacturing. Workers exposed to extreme heat frequently experienced an increased risk of injury due to impaired cognitive and physical abilities resulting from fatigue, dehydration, and heat stress. Young males, particularly in the construction and agricultural sectors, were consistently identified as the most vulnerable.

#### 3.2.2. Heat Stress/Heat Strain

Heat stress significantly reduced workers’ physical and cognitive performance. Common coping strategies included increasing water intake, adjusting work schedules, and taking regular breaks, mentioned in a total of 30 articles, e.g., [23,24,25,26]. Many workers still lacked adequate awareness and preparedness for dealing with heat stress, underscoring a critical need for enhanced education and improved workplace policies.

#### 3.2.3. Occupational Heat Stress/Illnesses/Injuries/Accidents

Occupational injuries related to heat were prevalent in labor-intensive sectors, including construction and agriculture, as mentioned in a total of 30 articles e.g., [9,16,20,34,35]. High temperatures were associated with increased accident rates, particularly among workers engaged in physically demanding tasks. The use of geographic and technological tools (like GIS and VR simulations) to map and prevent accidents was found to be beneficial in several studies.

#### 3.2.4. Construction Workers and Workplace Safety

Effective workplace safety practices included the provision of shaded rest areas, the availability of hydration stations, and the implementation of work–rest schedules, as mentioned in 40 articles, e.g., [1,38,39,40]. Despite recognized safety measures, actual workplace practices often fell short due to limited implementation or inadequate training of workers.

Table 3 shows that the most common finding across all the studies was a strong correlation between high temperatures and an increased risk of occupational injury. In contrast, factors such as hydration status, blood pressure, or pulse rate remained underexplored, suggesting the need for more physiological and biomonitoring studies. Heat conditions frequently compromised physical and mental performance, resulting in higher incidents of accidents, reduced productivity, and serious health risks. Despite clear evidence linking heat to occupational hazards, many workplaces lacked comprehensive heat stress management strategies. Practical preventive measures (such as rest breaks and hydration practices) were commonly recommended, yet often inadequately implemented. Significant gaps existed in the systematic management of occupational illnesses related to heat, the quantification of productivity loss, and the practical implementation of preventive measures at workplaces. Overall, the studies collectively emphasized the urgent need for more structured and consistently enforced occupational health standards, targeted education and training for workers, and greater research attention on less-studied topics, such as heat exposure, thermal stress, occupational illness, and productivity loss, to effectively mitigate heat-related workplace risks.

It is evident from the scoping review findings that there were regional variations between the studies. The studies conducted in North America and Europe predominantly focused on ambient temperature and workplace safety, whereas the studies conducted in Asia often emphasized humidity, WBGT, and physiological strain, highlighting climatic and occupational context differences. Moreover, the differences in methodological approach might also contribute to the variability in the study findings. While the majority of the studies used observational study designs, few incorporated personal exposure monitoring or longitudinal measurements, which may limit the potential causal inference. Future research is warranted to integrate multifactorial analyses to jointly investigate physiological, environmental, and workplace risk factors to illuminate the process of interaction among these risk factors across regions and worker populations as well.

## 4. Discussion

This scoping review, enriched by recent studies, reveals a complex and evolving understanding of extreme heat exposure risks in the construction industry. The synthesis of 42 peer-reviewed articles and additional contemporary research highlights the multifactorial nature of heat-related health outcomes, shaped by physiological, environmental, occupational, and policy dimensions. Based on this synthesis, we present evidence-based prevention guidelines and regulatory recommendations to address the critical gaps in current heat stress management practices.

### 4.1. Heat Exposure and Injury Risk: Comparative Insights

Elevated temperatures are consistently linked to increased occupational injury risk, with recent studies highlighting not only the role of extreme heat but also the compounding effects of humidity, solar radiation, and demographic vulnerabilities. The correlation between elevated temperatures and occupational injuries is well established. Fatima et al. (2021) found a 1% increase in injury risk per 1 °C rise in temperature, with heatwaves elevating risk by 17.4% [20]. This aligns with Binazzi et al. (2019), who reported a statistically significant increase in injury risk, particularly among younger males [35]. Amorim’s recent field study in Kansas City adds nuance, showing that even moderate temperatures (88 °F) led to internal body temperatures exceeding 100.4 °F in 43% of workers [55]. This suggests that humidity and solar radiation, not just temperature, critically influence physiological strain. Calkins et al. (2019) and Dong et al. (2019) emphasized demographic vulnerabilities, with younger, less experienced, and Hispanic workers facing higher risks [1,6]. Amorim’s study corroborated this, identifying roofers and cement masons as particularly vulnerable due to direct sun exposure and heat-retaining materials [55]. Our summary identifies particular worker cohorts requiring focused preventative interventions to mitigate their increased susceptibility to heat-related accidents. Enforcing obligatory acclimatization methods is crucial, necessitating 5–14 days of incremental heat exposure for new employees and those resuming work after absences exceeding one week, as heat tolerance rapidly declines without consistent exposure. Inexperienced workers, especially those under 25 years of age or with less than two months of employment, are at heightened danger and require vigilant supervision during heat events. Specific trades, such as roofing and cement masonry, necessitate additional safeguards; granting them 15 min rest intervals each hour when temperatures are above 85 °F recognizes their vulnerability to direct sunshine and heat-absorbing materials. During extreme heat days, the implementation of mandatory buddy systems is essential, pairing experienced workers with novices to guarantee ongoing peer support and the dissemination of knowledge regarding the signs of heat stress. High-risk tasks, such as roofing and concrete work, should be performed during cooler morning hours before 10 AM, in order to balance project requirements and noise limits. These tailored interventions acknowledge that heat vulnerability differs within the construction workforce and must be handled appropriately.

### 4.2. Physiological Monitoring and Health Outcomes

Physiological indicators, such as heart rate, core temperature, and hydration status, are central to understanding heat stress. Tracking heart rate, body temperature, and hydration can help spot heat stress early. Studies show many workers start their shifts dehydrated, even when water is available—pointing to the need for better hydration habits and health monitoring on the job. Kakamu et al. (2022) demonstrated that an elevated heart rate from rest is a reliable early marker of heat-related illness [17]. Amorim’s ingestible capsule study is the first in the U.S. to directly measure core temperature in construction workers, revealing widespread dehydration at the start of shifts despite water availability [55]. This finding echoes Al-Bouwarthan et al. (2020a), who found similar dehydration patterns in Saudi Arabia [25]. The convergence of these studies across regions underscores the need for pre-shift hydration protocols and continuous physiological monitoring.

The prevalent dehydration trends shown in various studies require thorough hydration and monitoring strategies that focus on both prevention and early identification of heat stress. Construction sites must do obligatory pre-shift hydration evaluations, utilizing urine color charts or specific gravity measurements to detect workers who commence their shifts in a dehydrated condition. To avert initial dehydration, workers must drink 500–750 mL of water two hours before the start of their shift, thereby establishing sufficient baseline hydration. During the workday, hourly hydration breaks must be instituted, requiring the drinking of a minimum of 250 mL of water to provide consistent fluid replacement that corresponds to sweat losses. Physiological monitoring thresholds must be explicitly established, necessitating obligatory rest intervals when a worker’s sustained heart rate surpasses 75% of their age-predicted maximum (calculated as 220 minus age, multiplied by 0.75) for over five minutes, thereby offering an objective standard for heat strain intervention. Construction sites should implement designated “heat stress stations” furnished with hydration resources, cooling zones, and physiological monitoring equipment, thereby establishing centralized facilities that facilitate compliance for all workers.

### 4.3. Wearable Technologies: Contrasts and Innovations

Existing heat stress assessment methodologies demonstrate fundamental constraints that compromise their utility in dynamic construction environments. Traditional approaches, including wet-bulb globe temperature (WBGT) measurements and worker symptom self-reporting, fail to provide real-time, individualized data necessary for proactive risk management [56]. These methods rely heavily on meteorological data that may not accurately represent specific workplace microclimates, local heat sources, or individual heat production rates [9]. Furthermore, current monitoring approaches inadequately account for individual variability in heat strain response, acclimatization status, and personal risk factors [57]. Recent advances in wearable technologies offer promising solutions. Saidi and Gauvin (2023) and Marinaccio et al. (2019) advocated for ECG-based sensors over traditional WBGT indices [41,43]. Amorim’s study used ingestible sensors, while Boston University’s C-HEAT project tested wearable fans and cooling towels, finding that workers preferred neck fans over cooling vests [55]. Kenzen’s arm-worn devices, tested in worksites, predicted heat stress 15–30 min before symptoms appear [58]. Compared to Amorim’s passive monitoring, Kenzen’s predictive analytics represented a proactive shift in heat stress management. Despite these innovations, barriers remain. It is noted that ethical concerns, integration challenges, and high costs are obstacles to widespread adoption. While wearable tech is advancing, its scalability and accessibility require further attention [59]. The advancement of wearable technologies demands a carefully planned implementation strategy that balances innovation with practical application. Organizations are encouraged to launch pilot wearable monitoring programs through a phased rollout, beginning with the highest-risk workers—such as roofers and concrete laborers—before expanding site-wide. This focused approach allows for refining protocols while prioritizing the most vulnerable groups. Establishing minimum technological standards is crucial, including real-time heart rate monitoring for all employees whenever the wet-bulb globe temperature (WBGT) exceeds 27 °C, ensuring essential physiological oversight during critical heat exposure periods. Standardized alert systems must be implemented to guarantee immediate supervisor notification when workers exceed safe physiological limits, enabling swift interventions to prevent heat-related illnesses. To overcome financial barriers to technology adoption, stakeholders should develop innovative cost-sharing models involving contractors, insurers, and regulatory bodies, recognizing that enhanced worker safety benefits all parties through reduced injury claims and improved productivity. Equally important is enforcing strict data privacy protections to prevent the misuse of physiological monitoring data for punitive purposes, addressing legitimate surveillance concerns and maintaining the trust that is vital for the program’s success.

### 4.4. Productivity Loss and Economic Impacts

Heat stress has measurable impacts on labor productivity, with micro- and macro-level evidence indicating significant economic consequences. Han et al. (2024) found that 60% of construction workers experienced productivity loss when the WBGT exceeded 28 °C [46]. This is supported by the Federal Reserve Bank of San Francisco, which projects a 5.4% reduction in U.S. capital stock and a 1.8% drop in annual consumption by 2200 due to heat-induced productivity loss [60]. These macroeconomic projections highlight the long-term implications of heat stress beyond individual health. Amorim’s study added micro-level evidence, showing that even mild heat stress disrupts work rhythms and increases fatigue [55]. To alleviate the significant economic repercussions highlighted in our analysis, construction firms could adopt flexible work schedules that respond directly to temperature conditions. When the WBGT exceeds 28 °C, a balanced 50% work and 50% rest schedule should be implemented, shifting to a more cautious 25% work and 75% rest ratio when temperatures rise above 30 °C. Governments and regulatory agencies should offer economic incentives, such as tax credits for firms investing in heat mitigation technologies and comprehensive training programs, to reduce implementation costs while enhancing worker safety. Project planning must incorporate reasonable timetable adjustments that account for expected heat-related productivity declines during summer, avoiding unrealistic deadlines that compromise safety. Moreover, contractors should be required to include heat stress management costs in their project proposals, ensuring that competitive bidding does not undermine essential safety measures. These integrated economic strategies recognize that protecting worker health and maintaining productivity are complementary, not conflicting, goals.

### 4.5. Gender and Psychosocial Dimensions

Gender differences in heat-related risks are underexplored. Curtis et al. (2022) found that tradeswomen reported higher stress and injury rates than men, with psychosocial exposures like discrimination and harassment contributing significantly [61]. Han et al. (2024) also noted that female/mixed-gender teams were more susceptible to productivity loss [46]. Another study found that women face more psychosocial hazards, while men encounter more physical risks [62]. These findings suggest that gender-sensitive interventions are essential, including tailored PPE, inclusive safety training, and supportive workplace cultures. The implementation of gender-inclusive safety protocols, including the provision of appropriately fitted personal protective equipment and cooling vests tailored for various body types, the establishment of confidential reporting systems for heat-related symptoms to mitigate stigma, the requirement of gender-inclusive heat safety training that addresses both physical and psychosocial stressors, and the creation of separate cooling/rest areas upon request, may enhance safety and comfort for all workers.

### 4.6. Policy Effectiveness and Regional Contrasts

The substantial economic burden of heat-related occupational injuries—both direct and indirect—warrants urgent attention from policymakers and industry leaders. Policy responses vary widely. Amorim’s study revealed that OSHA lacks a federal heat standard, relying on state-level regulations in California, Oregon, and Washington. Schinasi et al. (2024) found limited impact from Dallas County’s rest break ordinance, suggesting that enforcement and education are critical [49]. A meta-analysis showed that cooling vests and structured work–rest schedules are among the most effective interventions [63]. However, implementation remains inconsistent. The needs for more effective and practical heat stress prevention strategies and guidelines in construction are documented [64]. Regional heat action plans must be formulated to consider local climate trends, workforce demographics, and current infrastructure. Countries with established occupational health systems ought to incorporate mandatory reporting of heat-related illnesses and near-miss accidents into their existing surveillance frameworks, while those developing such systems should emphasize heat stress as a fundamental component. The establishment of certification programs for heat safety officers should be tailored to local project scales and economic conditions. We must modify these limits to align with the specific characteristics of the national construction industry and its regulatory capabilities. These suggestions establish a framework that can be tailored to various regulatory contexts while preserving fundamental protection requirements for construction workers worldwide. The implementation of gender-inclusive safety protocols, including the provision of appropriately fitted personal protective equipment and cooling vests tailored for various body types, the establishment of confidential reporting systems for heat-related symptoms to mitigate stigma, the requirement of gender-inclusive heat safety training that addresses both physical and psychosocial stressors, and the creation of designated cooling/rest areas upon request, may enhance the safety and comfort of all workers.

## 5. Conclusions

This scoping review highlights the complex nature of heat-related risks in the construction industry, which are influenced by a combination of physiological, occupational, environmental, and policy factors. The synthesis of 42 peer-reviewed articles demonstrates a consistent association between elevated temperatures and increased risk of occupational injuries, particularly among younger, less experienced, and vulnerable worker groups. The scoping review findings also reported productivity losses when the WBGT exceeded 28 °C. Integrating these results into policy or workplace practice frameworks includes improved hydration and cooling measures, recommending temperature-based work–rest cycles, and adjustments to work hours during extreme heat. Therefore, incorporating WBGT thresholds into occupational safety standards to guide employers in preventing productivity loss and heat-related illnesses is warranted.

Moreover, traditional heat monitoring methods are insufficient, while wearable technologies, especially ECG-based sensors, offer promising solutions for real-time physiological assessment. Despite technological advancements, integration challenges and equity concerns persist. Heat exposure and exposure–response relationships also impact productivity and incur economic costs, thereby further amplifying the urgency for effective interventions. Gender specific and psychosocial stressors highlight the need for inclusive, tailored, individualized safety measures. Regional disparities and inconsistent policy enforcement indicate that federal standards and evidence-based prevention strategies are essential. As ambient temperature intensifies, the construction industry must evolve from reactive to proactive heat stress management. Integrating scientific evidence, technological innovation, and policy reform is essential to safeguarding worker health and productivity.

Beyond the current limitations, future research should prioritize longitudinal studies that explore the long-term health effects of repeated occupational heat exposure throughout workers’ lives, especially focusing on potential links to chronic kidney disease and cardiovascular conditions. There remain significant gaps in our knowledge regarding the cost-effectiveness of different intervention strategies, the application of implementation science to overcome barriers in small- to medium-sized construction firms, and the development of predictive biomarkers to identify individuals most vulnerable to heat stress. Additionally, research should investigate the best recovery methods after heat exposure, how heat stress interacts with other workplace hazards, such as chemicals, noise, and vibration, and the effectiveness of culturally tailored training programs for diverse, multilingual construction crews. Addressing these areas will help move from merely identifying risks to delivering evidence-based, practical solutions.

## Figures and Tables

**Table 1:** Absolute and relative frequency of various article characteristics (*n* = 42).

Characteristics	Frequency	%
Country of Origin		
USA	10	23.81
Canada	1	2.38
Ireland	1	2.38
Italy	4	9.52
Spain	1	2.38
Slovenia and Greece	1	2.38
Australia	3	7.14
China	2	4.76
Taiwan	1	2.38
Thailand	1	2.38
Malaysia	1	2.38
India	1	2.38
Vietnam	1	2.38
Japan	3	7.14
Saudi Arabia	3	7.14
United Arab Emirates	1	2.38
Qatar	1	2.38
Canada, USA, Australia, China, Spain, Italy and Thailand	1	2.38
Canada, USA, Australia, China, Spain, Italy	1	2.38
India, China, Thailand, Iran, Saudi Arabia, Australia, Italy, and Sweden	1	2.38
Canada, Australia, China, Italy, Spain, and the USA	1	2.38
North America, Europe, and Asia	1	2.38
Africa, Middle East and Arid Asia, Australia and Pacific Islands, Asia, Europe, and the Americas	1	2.38
Publication Year		
2024	9	21.43
2023	8	19.05
2022	4	9.52
2021	6	14.29
2020	4	11.90
2019	11	26.20
Study Type		
Review	8	19.05
Observational Study	21	50.00
Experimental Study	6	14.29
Empirical Study	5	11.90
Clinical Case Report/Case Study design	1 of Each	4.76

**Table 3:** Key risk factors discussed in the included studies.

Key Risk Factors Discussed	Total Number of Studies
Weather Factors	
Temperature/extreme heat/heat exposure	36
Heat stress/heat strain/thermal stress	30
Wet-bulb globe temperature (WBGT)	10
Humidity	9
Physiological Factors	
Skin/body/oral/auditory canal temperature	9
Hydration status	4
Heart rate	10
Pulse rate	2
Blood pressure	3
Individual factors	
Age	10
Gender	4
Heat-related illness	33
Occupational heat stress/illness/injuries/accidents	28
Workplace factors	
Construction/construction workers	37
Workplace safety	40

## Data Availability

No new data were created or analyzed in this study. Data sharing is not applicable to this article.
